# Advances in treatment of alveolar soft part sarcoma: an updated review

**DOI:** 10.1093/jjco/hyad102

**Published:** 2023-08-25

**Authors:** Tomohiro Fujiwara, Toshiyuki Kunisada, Eiji Nakata, Kenji Nishida, Hiroyuki Yanai, Tomoki Nakamura, Kazuhiro Tanaka, Toshifumi Ozaki

**Affiliations:** Department of Orthopaedic Surgery, Okayama University Graduate School of Medicine, Dentistry, and Pharmaceutical Sciences, Okayama, Japan; Department of Orthopaedic Surgery, Okayama University Graduate School of Medicine, Dentistry, and Pharmaceutical Sciences, Okayama, Japan; Department of Orthopaedic Surgery, Okayama University Graduate School of Medicine, Dentistry, and Pharmaceutical Sciences, Okayama, Japan; Department of Pathology, Okayama University Hospital, Okayama, Japan; Department of Pathology, Okayama University Hospital, Okayama, Japan; Department of Orthopaedic Surgery, Mie University, Tsu, Japan; Department of Advanced Medical Sciences, Oita University, Yufu, Japan; Department of Orthopaedic Surgery, Okayama University Graduate School of Medicine, Dentistry, and Pharmaceutical Sciences, Okayama, Japan

**Keywords:** alveolar soft part sarcoma, surgery, chemotherapy, targeted therapy, immunotherapy

## Abstract

Alveolar soft part sarcoma is a rare neoplasm of uncertain histogenesis that belongs to a newly defined category of ultra-rare sarcomas. The neoplasm is characterized by a specific chromosomal translocation, der (17) t(X; 17)(p11.2;q25), that results in *ASPSCR1–TFE3* gene fusion. The natural history of alveolar soft part sarcoma describes indolent behaviour with slow progression in deep soft tissues of the extremities, trunk and head/neck in adolescents and young adults. A high rate of detection of distant metastasis at presentation has been reported, and the most common metastatic sites in decreasing order of frequency are the lung, bone and brain. Complete surgical resection remains the standard treatment strategy, whereas radiotherapy is indicated for patients with inadequate surgical margins or unresectable tumours. Although alveolar soft part sarcoma is refractory to conventional doxorubicin-based chemotherapy, monotherapy or combination therapy using tyrosine kinase inhibitors and immune checkpoint inhibitors have provided antitumor activity and emerged as new treatment strategies. This article provides an overview of the current understanding of this ultra-rare sarcoma and recent advancements in treatments according to the clinical stage of alveolar soft part sarcoma.

## Teaser

Alveolar soft part sarcoma (ASPS), a newly defined ultra-rare sarcoma, is characterized by specific gene fusions including *ASPSCR1–TFE3*. While complete surgical resection remains a standard treatment, tyrosine kinase inhibitors and immune checkpoint inhibitors have emerged as new treatment strategies for ASPS.

## Introduction

Soft-tissue sarcomas (STSs) are a heterogeneous group of rare tumours that arise in mesenchymal tissues and comprise more than 80 histological entities (1,2). Alveolar soft part sarcoma (ASPS) is a very rare sarcoma of uncertain histogenesis that accounts for <1% of all soft-tissue sarcomas and belongs to a newly defined category of ultra-rare sarcomas (3). ASPS was originally described in 1952 as ‘malignant myoblastoma’ or ‘granular-cell myoblastoma’ and defined histologically as consisting of cell ‘nests’ loosely arranged along connective tissue containing sinusoidal vascular channels lined by flattened endothelium with characteristic intracytoplasmic rod-shaped crystals (4). These nests separated by capillaries appeared to resemble lung alveoli, which is the origin of the name of this sarcoma (4,5). ASPS is characterized by a specific translocation, der (17)t(X;17)(p11.2;q25), which results in *ASPSCR1–TFE3* gene fusion (6). Although ASPS is refractory to conventional cytotoxic chemotherapy and radiotherapy, recent reports have discussed the possible benefits of targeted therapy, such as antiangiogenic drugs and immune-stimulating therapy (7). This review article aimed to summarize the clinicopathological characteristics of ASPS and to update current views regarding its diagnosis and treatment.

## Epidemiology

ASPS is an ultra-rare sarcoma with an incidence rate of only one diagnosis per 10 million population per year, accounting for 0.2–0.9% of all soft-tissue sarcomas (8). From the Bone and Soft Tissue Tumor Registry (BSTTR) database in Japan, diagnosis of ASPS was recorded in 51 (2.1%) of 2474 patients with soft-tissue sarcomas registered from 1985 to 1994 (9). ASPS most commonly occurs in adolescents and young adults (age range, 15–39 years), with a slight predominance in females. In the Surveillance, Epidemiology, and End Results (SEER) database, the patients had a median age of 25 (range, 1–78) years, 72% were <30 years old and 58% were females (2,10).

## Clinical presentation

ASPS presents asymptomatically as a slow-growing tumour (4,11) and commonly arises in deep soft tissues of the extremities (61%), trunk (20%), head and neck (9%), and internal organs (8%) (2,10). Uncommon sites include bone (12,13), brain (14,15), orbit (16), tongue (17), lung (18), mediastinum (19,20), bladder (21), prostate (22), uterus (23) and vagina (24). Because of the lack of associated symptoms or functional impairment, patients with ASPS often present with metastatic disease (4). At presentation, 28% of patients have localized disease and 72% have metastatic disease in the BSTTR database (25), which was similar to the finding in the National Cancer Data Base (NCDB) in the USA (26). The most common metastatic sites are the lung, bone, brain and liver (3). In an analysis of the BSTTR database, 45% (*n* = 13/34) of patients with localized ASPS developed distant metastases in the lung (*n* = 12, 92%) and brain (*n* = 2, 15%), whereas the sites of metastasis in patients with stage 4 disease at diagnosis (*n* = 86) were lung (*n* = 85; 99%), bone (*n* = 12; 14%) and brain (*n* = 9; 11%) (25).

## Radiological features

**Figure 1:**
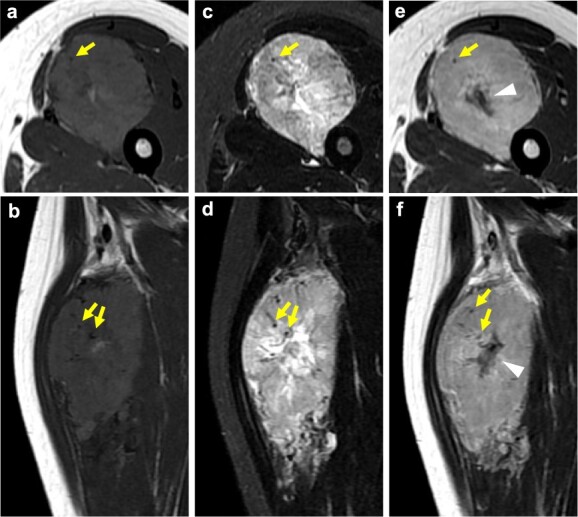
Radiological features of alveolar soft part sarcoma. Magnetic resonance (MR) images of a tumour involving the quadriceps femoris in a 19-year-old female. (a, b) Axial (a) and sagittal (b) T1-weighted MR images. The tumour signal intensity is higher than the muscle signal intensity. Peritumoral abnormal vessels (arrows) can be seen in the tumour. (c, d) Axial (c) and sagittal (d) T2-weighted MR images. The tumour shows high signal intensity, which was not affected by fat suppression. Peritumoral abnormal vessels (arrows) can be seen. (e, f) Axial (e) and sagittal (f) gadolinium-enhanced T1-weighted MR images. The tumour shows a central area without enhancement corresponding to the necrotic area (marks). Peritumoral abnormal vessels (arrows) can be seen.

On computed tomography (CT) imaging, ASPS is observed as an enhancing mass lesion with prominent feeding vessels (27). Prominent intratumoural blood vessels are observed on contrast-enhanced CT (28). CT is also used to detect distant metastases to the brain, lung and bone for initial tumour staging. On magnetic resonance imaging (MRI), ASPS typically shows high signal intensity in T1- and T2-weighted imaging and features internal and external multilobulated signal changes (27). Crombé et al. analysed MRI features in 25 patients with ASPS and concluded that deep-seated tumours presenting with mainly high signal intensity on T1-weighted imaging, an absence of fibrotic component, ill-defined margins without aponeurotic extension, and more than five central and peripheral flow-voids are very likely to be ASPS (29). The mass shows intense enhancement and multiple peritumoral and intratumoural tortuous signal voids on contrast-enhanced MR images (27) (Fig. 1). ASPS may be misdiagnosed as haemangioma or arteriovenous malformation, which occur in the same age group (29). Final diagnosis, however, is based on tissue biopsy, as for all other subtypes of soft-tissue sarcoma.

## Histopathological features

**Figure 2:**
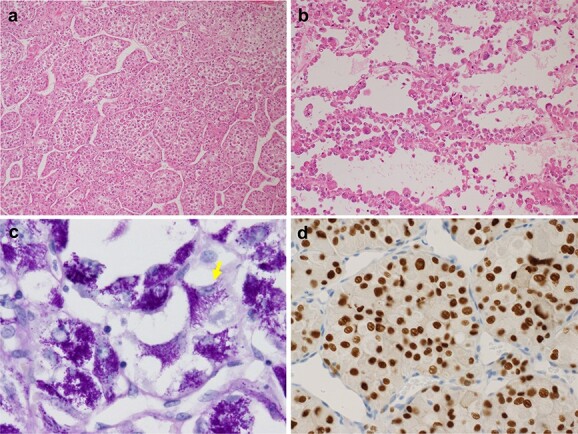
Histopathological features of ASPS. (a) Typical organoid nests composed of large eosinophilic tumour cells. (b) The prominent pseudoalveolar growth pattern of ASPS. (c) PAS staining showing the varying amounts of cytoplasmic glycogen and rod-shaped crystals (arrow). (d) Nuclear TFE3 immunostaining.

The microscopic figure is uniform. ASPS comprises organoid nests outlined in sinusoidal vessels (Fig. 2a) (29–31). The tumour nests show central degeneration and loss of cellular cohesion, resulting in a characteristic pseudoalveolar pattern (Fig. 2b) (32,33). Tumour cells have a well-defined border, abundant eosinophilic cytoplasm, round vesicular nucleus and prominent nucleolus (32,33). Histochemically, PAS stain shows varying amounts of cytoplasmic glycogen and characteristic rod-shaped crystals (Fig. 2c) (34). This tumour is characterized by aberrations in *TFE3* genes, showing nuclear immunoreactivity for this protein product in most cases (Fig. 2d) (33,35,36).

## Molecular genetic features

ASPS is characterized by a specific chromosomal alteration, der (17)t(X:17)(p11:q25), resulting in the fusion of the *TFE3* transcription factor gene (from Xp11) with alveolar soft part sarcoma critical region 1 (*ASPSCR1*), also known as alveolar soft part sarcoma locus at 17q25 (6,33). Detection of this fusion transcript, *ASPSCR1–TFE3*, through real-time polymerase chain reaction or fluorescence *in situ* hybridization for *TFE3* rearrangements are considered useful methods for diagnosis (33,37). This fusion protein acts as an aberrant transcription factor resulting in activation of the MET signalling pathway believed to promote angiogenesis and cell proliferation (2,33). Although the presence of *ASPSCR1–TFE3* fusion is highly specific to ASPS, the same gene fusion is also seen in a small but unique subset of renal cell carcinomas (2). Recently, novel alternative rearrangements, including *HNRNPH3–TFE3*, *DVL2–TFE3* and *PRCC–TFE3* gene fusions, have been identified, highlighting genetic diversity in ASPS (3,38).

## Natural history and prognostic factor

ASPS is characterized by its indolent behaviour with slow progression (3). However, the metastatic potential appears to be greater for ASPS than for other soft-tissue sarcomas; patients often present with a metastatic stage at the time of diagnosis (37,39). The NCDB (USA) retrospective study included 293 patients ≥18 years who were diagnosed between 2004 and 2015, among whom 59% (*n* = 172/293) had a metastatic stage (26). Patients with head and neck tumours were least likely (40%) to present with distant disease (26). In BSTTR (Japan), 34 (28%) patients presented with localized disease (stage II, 13%; stage IIIA, 11%; stage IIIB, 4% by American Joint Committee on Cancer staging) at diagnosis and 86 (72%) with metastatic disease (25). Patients who were >25 years old, had deep-seated tumours and tumours >5 cm were more likely to have metastatic disease (25). The common metastatic sites are the lung, brain, bone and liver (3). Of note, ASPS is characterized by higher rates of brain metastasis than other STSs. In the BSTTR study, distant metastases to the lung and brain developed in 12 (35%) and 2 (6%) of 34 patients with localized disease at diagnosis, respectively, and the sites of metastasis in 86 patients with metastatic disease at diagnosis were the lung in 85 patients (99%), bone in 12 (14%) and brain in 9 (11%) (25). Therefore, intracranial imaging should be added to routine imaging studies, as mentioned in the current clinical practice guidelines (1,40,41).

The prognostic factors previously reported in the literature include age at presentation, tumour size, bone involvement and presence of metastasis at diagnosis (Table 1). For localized ASPS, the 5-year overall survival (OS) rate is 60–88%, which was reported by previous studies, excluding those with <30 patients (Table 1). A survival rate of 60% reported in 1989 by Lieberman et al. for patients with localized disease appears to have improved slightly to 73–87% for that group. This might be because of improvements in surgical techniques; Hagerty et al. reported in the NCDB study that the margin status (positive/negative) was univariable associated with OS. For metastatic ASPS, the 5-year OS rate is 20–62%, as reported by previous studies, excluding those with <30 patients (Table 1) (10,25,26,42–45). The 5-year OS rates reported before 2010 were 22% by Lieberman et al. (43), 20% by Portea et al. (45) and 46% by Ogose et al. (44), whereas the rate was 61% by Flores et al. (42), 46% by Hagerty et al. (26) and 62% by Fujiwara et al. (25) (Table 1). The improvement in survival outcome might be because of the introduction of targeted therapy. In the BSTTR analysis, a comparison of survival outcomes before and after the approval of pazopanib was performed, and a trend toward superior disease-specific survival (DSS) was observed in patients who had a diagnosis and/or treatment for metastatic ASPS after 2012 (5-year DSS, 65%) than before 2012 (5-year DSS, 58%) when the clinical use of pazopanib was approved in Japan (25). Further exploration of targeted agents, immunotherapy and their combination may further improve survival outcomes.

**Table 1:** Oncologic outcomes and prognostic factors in localized and metastatic alveolar soft part sarcoma

Author (Refs)	Year	Number of patients			5-Year survival			Prognostic factor
		Overall	Localized	Metastatic	Overall	Localized	Metastatic	
Lieberman et al. (43)	1989	91	69	22	57%	60%	22%	Age, metastasis
Casanova et al. (46)	2000	19 (paediatric)	15	4	80%	91%	NA	Size
Portea et al. (45)	2001	74	22	52	47%	88%	20%	Metastasis
Ogose et al. (44)	2003	57	20	37	56%	81%	46%	Metastasis, size, bone involvement
Ogura et al. (56)	2012	26	10	16	64%	100%	37%	Size, metastasis
Wang et al. (10)	2016	251	118	108	56%	81%	41% (surgery+) 10% (surgery−)	Age, size, trunk, metastasis, no treatment, RT without surgery
Brennan et al. (49)	2018	22 (paediatric)	20	2	100%	100%	100%	NA
Flores et al. (42)	2018	69 (paediatric)	31	38	72%	87%	61%	Age, sex, metastasis
Hagerty et al. (26)	2020	293 (NCDB)	83	172	NA	73% (surgery+)	46% (surgery+)	Metastasis, size, margin, multimodal therapy, hospital volume
Fujiwara et al. (25)	2022	120 (BSTTR)	34	86	68%	86%	62%	Metastasis

*Abbreviations*: NCDB, National Cancer Database; BSTTR, Bone and Soft Tissue Tumor Registry; RT, radiotherapy; NA, not available.

## The management of localized ASPS

### Surgery

Surgical resection is the standard treatment for other subtypes of soft-tissue sarcoma. The standard surgical procedure is complete resection with wide margins. In a multi-institutional, retrospective study from the Japanese Musculoskeletal Oncology Group (JMOG), Ogose et al. reported that the rates of local recurrence following surgery alone were 0% (*n* = 0/36) with wide margins, 57% (*n* = 4/7) with marginal margins and 100% (*n* = 1/1) with intralesional margins (44). Complete resection may be curative in some patients, but metastases are common with long-term follow-up after resection of the primary tumour (37). In the BSTTR database study, the 5-year metastasis-free survival was 19% after wide resection for localized ASPS (25).

### Radiotherapy

There is no consensus on the current role of adjuvant radiotherapy (RT) because of the lack of evidence of improved local control and survival rates. In the SEER database analysis, Wang et al. reported that OS was better for surgery plus RT (*n* = 54) than for surgery alone (*n* = 64) in patients with localized ASPS (10). Although patients with a larger tumour size (>5 cm) were more likely to receive RT (10), it was unclear if the indication of RT was determined on the basis of the surgical margins of the primary tumour. Casanova et al. suggested the use of RT in patients with inadequate surgical margins (46), but they did not recommend the use of RT in all children to prevent delayed morbidity since local control is probably unnecessary if the tumour is adequately excised (46). In the JMOG study, patients who underwent marginal excision and RT (*n* = 3) had no local recurrence, whereas local recurrence occurred in 4 (57%) of 7 patients who underwent marginal excision alone (44). Patients who underwent wide excision (*n* = 36) and amputation with wide margins (*n* = 2) had no local recurrence (44). Although these data suggest a clinical benefit for adjuvant RT with inadequate surgical margins, further investigation with a larger cohort of patients is necessary, which should be considered to assess the role of surgical margins and systemic treatments.

Definitive RT using carbon-ion RT may be one treatment option for unresectable ASPS. Nakao et al. reported a case of a 9-year-old girl with localized ASPS arising in the upper third of her vagina (47). Carbon-ion RT with 67.2 Gy in 16 fractions was delivered to the residual tumour adhered to the posterior pubis after partial resection of the tumour, which decreased gradually in size without tumour recurrence over 20 months (47). Okamoto et al. reported a case of a 24-year-old woman with unresectable ASPS arising in the right pelvic lesion and right lower leg (48). Carbon-ion RT with 67.2 Gy in 16 fractions was delivered to the pelvic tumour followed by anti-programmed cell death protein 1 (PD-1) antibody (pembrolizumab). Both the irradiated pelvic tumour and nonirradiated leg tumour decreased remarkably in size (80%), which was confirmed on the MRI taken 10 months after carbon-ion RT (48). Long-term efficacy of carbon-ion RT is awaited.

### Chemotherapy

The efficacy of adjuvant chemotherapy on ASPS reportedly has been ineffective to date. Conventional anthracycline-based chemotherapy is largely inactive, with response criteria in solid RECIST rates <10% (37,45,49,50). In the JMOG study by Ogose et al., 21 patients with primary tumours underwent neoadjuvant chemotherapy; none of 14 patients who received systemic chemotherapy based on doxorubicin-, ifosfamide- and cisplatin-based regimens showed a clinical response of stable disease (SD, *n* = 12) or progressive disease (PD, *n* = 2), and 5 (71%) of 7 patients who underwent intra-arterial chemotherapy mainly with cisplatin showed no clinical response (44). In the EpSSG NRSTS 2005 study, 4 of 22 patients received chemotherapy with ifosfamide and doxorubicin, but there were no clinical responses (49). In a review of the published literature by Reichardt et al., the response to first-line chemotherapy (anthracycline alone, anthracycline plus ifosfamide and others) in 68 patients was PD in 51%, SD in 41%, partial response (PR) in 3% and complete response (CR) in 4% (50). Although these studies involved small series and it is necessary to collect more data from systematic analyses and clinical trials, standard regimens of cytotoxic chemotherapy agents as adjuvants appear to have no clinical benefit.

## The management of advanced/metastatic ASPS

### Surgery

In patients with advanced/metastatic ASPS, the effect of surgery on the primary lesion is controversial because of conflicting results. In the BSTTR database study, surgical resection of the primary site did not affect DSS; the 5-year DSS for the surgery (*n* = 57) and no-surgery (*n* = 29) subgroups were 68% and 51%, respectively (*p* = 0.559) (25). Contrarily, in the SEER database study by Wang et al., survival was significantly better for patients who underwent surgery for the primary lesion than for those without surgery; the 5-year OS rates for the surgery (*n* = 61) and no-surgery (*n* = 44) subgroups were 41% and 10%, respectively (*p* < 0.001) (10). Of note, in the NCDB study by Hagerty et al., patients with metastatic ASPS who underwent surgical resection of the primary tumour had longer OS (median OS: 48 months) than an identically selected population of patients with common histological sarcoma subtypes, including synovial sarcoma (median OS: 21 months), liposarcoma (median OS: 18 months), rhabdomyosarcoma (median OS: 11 months) and desmoplastic small round cell tumour (median OS: 28 months) (26). Since these survival outcomes differ among the databases in Japan and the USA, further prospective analysis with international collaborations would clarify the effect of surgical resection of the primary site in patients with advanced/metastatic ASPS.

Resectable metachronous (disease-free interval ≥ 1 year) lung metastases of soft-tissue sarcoma without extrapulmonary disease are managed with metastasectomy as standard treatment (1,51). However, studies investigating the effect of metastasectomy of ASPS are limited, probably because the incidence of oligometastatic ASPS is rare. In the BSTTR database study, the surgical resection of the metastatic site did not affect survival outcome: the 5-year DSS rates in patients with (*n* = 11) and without (*n* = 75) metastasectomy were 67% and 62%, respectively (*P* = 0.143) (25). Zhang et al. investigated 1184 patients with STS having metastasis at diagnosis and reported that surgery for metastasis was an independent factor associated with better survival (52). Although this was confirmed for a group of seven common histological subtypes (undifferentiated pleomorphic sarcoma, leiomyosarcoma, synovial sarcoma, myxoid liposarcoma, ASPS, malignant peripheral nerve sheath tumour and dedifferentiated liposarcoma), the survival benefit of metastasectomy in patients with ASPS remained unclear (52). Kodama et al. reported the clinical course of 4 patients who underwent aggressive excision of multiple metastases from ASPS. These patients underwent surgery of the primary tumour, followed by 8 pulmonary surgeries that excised 333 metastatic tumours (53). Although 3 of 4 patients died of tumour progression 40, 46 and 68 months after surgery of the primary tumour, 1 patient had been alive for 98 months after excision of the primary lesion (53). Of note, this was reported in 1997 when targeted therapy was not available, and the decision-making for metastasectomy should be multidisciplinary considering the accumulating evidence of currently available systemic agents.

### Radiotherapy

For the brain metastasis of ASPS, gamma-knife radiosurgery has been a reasonable option for local control. Flannery et al. reported satisfactory results in 21 patients who underwent gamma-knife stereotactic radiosurgery for intracranial sarcomatous metastases, including ASPS (*n* = 2) (55). The local control rate was 88%, the median survival after diagnosis was 16 months and the 1-year survival rate was 61% (55). Ogura et al. described satisfactory local control in 4 patients with brain metastasis of ASPS who underwent gamma-knife radiosurgery, with a median progression-free survival of 12 (range, 9–30) months (56). Lim et al. suggested the use of gamma-knife stereotactic radiosurgery with a single-dose ≥25 Gy for all brain metastases of ASPS. For large (>1.5 cm^3^) brain metastases of ASPS, all tumours treated with a low dose (<25 Gy) recurred, requiring surgical removal within 2 months following stereostatic radiosurgery, whereas the large tumour treated with a high dose (≥25 Gy) recurred after 11 months (57). For small (≤0.5 cm^3^) brain metastases of ASPS, 5 of 6 tumours treated with high doses ≥25 Gy were controlled, whereas the remaining tumour required additional treatment (57). Palliative whole-brain RT has been administered for several cases with multiple brain metastases, but the prognoses of these patients have been poor (55,58).

For the lung metastasis of ASPS, whole-lung irradiation is generally not performed as for other subtypes of STS except Ewing sarcoma. Strategies combining metastasectomy and RT may be used; a case report indicated the use of hyperfractioned local RT with a total dose of 44.8 Gy (2 × 1.6 Gy daily) following pulmonary metastasectomy (59).

### Chemotherapy

The standard treatment for patients with advanced/metastatic soft-tissue sarcomas is systemic chemotherapy with doxorubicin. For advanced/metastatic ASPS, however, previous studies have shown limited efficacy of doxorubicin-based chemotherapy. In a series from MD Anderson Cancer Center, 26 patients with metastatic ASPS at diagnosis were treated with systemic chemotherapy; doxorubicin-based chemotherapy was used in 17 (65%) of 26 patients (median, 4 cycles) (45). The majority of patients treated with chemotherapy (58%) developed disease progression and no partial or minor responses were noted (45). In the BSTTR analysis, administration of systemic therapy (conventional cytotoxic chemotherapy, 29%; targeted therapy, 40%; conventional cytotoxic chemotherapy + targeted therapy, 23%; others, 8%) did not affect survival outcomes in patients with metastatic ASPS (25). Among these patients, patients who received doxorubicin-based regimens had significantly inferior DSS; the 5-year DSS rates were 39% and 75% in patients with and without doxorubicin-based chemotherapy regimens, respectively (25). Patients who did not receive doxorubicin-based regimens were mostly treated with targeted agents (25). Because of the nature of drug resistance and oncogenic molecular pathways, several targeted agents have been tested and proven to be more beneficial than conventional cytotoxic chemotherapy, which are summarized in Table 2.

**Table 2:** Outcomes of monotherapy and combination therapy with targeted therapy and immunotherapy in alveolar soft part sarcoma

Drug	Phase	No. of patients	Outcome	Author (refs)	Year
***Targeted therapy***					
Pazopanib	Retrospective	30	1 CR, 7 PR, 17 SD, 4 PD, 1 NE; median PFS, 13.6 mo; PFS at 1 y, 59%	Stacchiotti et al. (64)	2018
	II (NCT02113826)	6	1 PR, 5 SD; median PFS, 5.5 mo; 6-mo PFS, 50%	Kim et al. (62)	2019
Sunitinib	Retrospective	9	5 PR, 3 SD, 1 PD; median TTP, 17 mo	Stacchiotti et al. (54)	2011
	Retrospective	14	4 PR, 10 SD; median PFS, 41 mo; 1-y OS, 90%; 4-y OS, 60%	Li et al. (67)	2016
	Retrospective	15	6 PR, 8 SD, 1 PD; median PFS, 19 mo; mOS, 56 mo; 5-y OS, 49%	Jagodzinska-Mucha et al. (66)	2017
Cediranib	II (00942877)	43	15 PR (ORR, 35%), 26 SD (ORR, 60%); 24-wk DCR, 84%	Kummar et al. (71)	2013
	II (NCT00942877)	7	ORR (24 wks), 35%; DCR (24 wks) 84%	Cohen et al. (89)	2019
	II (NCT01337401)	36 vs 16 (placebo)	Median percent change in diameter, −8.3% (cediranib) vs 13.4% (placebo) Median PFS, 10.1 mo (cediranib) vs 4.9 mo (placebo)	Judson et al. (70)	2019
Crizotinib	II (NCT01524926)	45	MET-positive: 1 PR, 35 SD, 1-y PFS 37.5%, 1-y OS 97.4% MET-negative: 1 PR, 3 SD, 1-y PFS 50%, 1-y OS 75%	Schoffski et al. (74)	2018
Cabozantinib	II (NCT01755195)	8	2 PR (ORR, 25%); 6-mo PFS, 71.4%	O’Sullivan Coyne et al. (75)	2022
Bevacizumab	Retrospective	1	Reduction of pulmonary and cerebral metastases	Azizi et al. (77)	2006
Sorafenib	Retrospective	1	SD, time on study, +67 weeks, still on study	George et al. (79)	2009
Dasatinib	II (NCT00464620)	12	1 PR (ORR 8%), median PFS, 11 mo; 6-mo PFS, 62%	Schuetze et al. (80)	2017
Tivantinib	II (NCT00557609)	27	21 SD, 5 PD, 1 NE; DCR, 78%; median PFS, 5.5 mo; 1-y OS, 84%; 2-y OS, 70%	Wagner et al. (81)	2012
Trabectedin	Retrospective	23	1 PR, 13 SD, 9 PD; median PFS, 3.7 mo; PFS at 1 y, 13%; median OS, 9.1 mo	Stacchiotti et al. (64)	2018
***Immunotherapy***					
ICIs	Retrospective	4	2 PR, 2 SD	Groisberg et al. (83)	2017
Pembrolizumab (anti-PD-1)	Retrospective	5	1 PR, 1 SD, 2 NA	Liu et al. (90)	2021
Nivolumab (anti-PD-1)	Retrospective	1	PD after 3 mo	Kuo et al. (84)	2016
	Retrospective	1	PD after 4.5 mo	Paoluzziri et al. (91)	2016
Nivolumab + ipilimumab (anti-CTLA-4)	Retrospective	1	PR (irRECIST, −51% from baseline)	Conley et al. (85) (84)	2018
Atezolizumab (anti-PD-L1)	Retrospective	1	PD after 6 doses but detected nonviable cells in the resected specimens	Vander Jagt et al. (86)	2018
GB226 (anti-PD-1)	II (NCT03623581)	37	ORR, 37.8%; DCR, 86.5% (RECIST), 91.9% (irRECIST); median PFS, 9.9 mo	Shi et al. (92)	2020
Durvalumab (anti-PD-L1) + tremelimumab (anti-CTLA-4)	II (NCT02815995)	10	ORR, 40% (irRECIST), 50% (irRC); 2 CR	Somaiah et al. (87)	2022
***Combination therapy***					
Sunitinib + nivolumab	Ib/II (NCT03277924)	7	4 PR (57%)	Matin-Broto et al. (88)	2020
Axitinib (anti-VEGF) + pembrolizumab	II (NCT02636725)	11	6 PR (54.5%), 2 SD (18%); 3-mo PFS, 72.7%	Wilky et al. (76)	2019

*Abbreviations*: PR partial response; SD, stable disease; PD, progressive disease, NE, not evaluable; NA, not available; DCR, disease control rate (PR + SD); PFS, progression-free survival; ICI, immune checkpoint inhibitor; irRECIST, immune-related response evaluation criteria in solid tumors; irRC, immune-related response criteria.

## Advances in treatment

### Targeted therapy

On the basis of the angiogenic properties of ASPS, several clinical trials have investigated various inhibitors of angiogenesis. To date, pazopanib and sunitinib have been recommended as preferred regimens for ASPS.

Pazopanib is a small-molecule tyrosine kinase inhibitor (TKI) exhibiting a selective activity against VEGF receptors (60). In a phase III study of metastatic STS (PALETTE study), the median progression-free survival (PFS) in patients receiving pazopanib was improved to 4.6 months compared with 1.6 months in those receiving placebo (61). For metastatic ASPS, a phase II study demonstrated that 1 of 6 patients enrolled achieved a PR, and 5 showed SD (62). The median PFS was 5.5 months, and the 6-month PFS rate was 50% (62). A retrospective study using the BSTTR database demonstrated that the median survival period in patients who received systemic therapy, including pazopanib, was 70 months (25). A trend towards improved survival was observed after 2012 when pazopanib was approved for metastatic STS in Japan (25). The mechanisms underlying the response to pazopanib in ASPS remain under evaluation. The antiangiogenic effect of pazopanib may target the peculiar vasculature of ASPS, sustained by the translocation-related activation of the lactate pathway in the tumour microenvironment (63,64). Kim et al. analysed the transcriptome of ASPS before and after pazopanib treatment; the top differentially expressed genes were related to angiogenesis and signalling pathways such as mitogen-activated protein kinase (MAPK), phosphoinositide 3-kinase (PI3K) and wingless-type MMTV integration site family (WNT) (62). These data indicated that pazopanib may modulate multiple signalling pathways in a simultaneous manner in ASPS.

Sunitinib has also shown promising efficacy for metastatic ASPS, although this agent is not approved for STSs in Japan. As a summary of multiple studies, administration of sunitinib demonstrated PR in 19 patients and SD in ≤24 of 46 evaluable patients (37,54,65–68). In addition, neoadjuvant use of sunitinib for primarily unresectable ASPS provided a change of complete surgical resection (67).

Other targeted drugs, including cediranib (69–72), crizotinib (73,74), cabozantinib (75), axitinib (76), bevacizumab (77,78), sorafenib (79), dasatinib (80) and tivantinib (81), have also shown therapeutic advantages in patients with advanced/metastatic ASPS (Table 2).

Trabectedin binding to the minor groove of DNA and blocking of DNA-repair machinery has also been approved for advanced/metastatic STSs. This agent was proven to be effective in translocation-related sarcomas (82). Stacchiotti et al. retrospectively reviewed the efficacy of trabectedin in 23 patients with ASPS, which revealed limited activity; 1 PR, 13 SDs and 9 PDs were observed, with a median PFS of 3.7 months and a median OS of 9.1 months (64).

### Immunotherapy

Immunotherapy is a promising area of drug development for ASPS. Immune checkpoint inhibitors (ICIs) such as anti-PD-1, anti-programmed death-ligand 1 (PD-L1) and anti-cytotoxic T-lymphocyte antigen 4 (CTLA-4) have been tested in patients with ASPS, although the number of patients has been limited (Table 2).

In the NCCN guideline, pembrolizumab (anti-PD-1 inhibitor) is recommended for treating ASPS. This recommendation is based on the results of the retrospective study by Grossberg et al., which studied 50 patients with advanced sarcoma who were referred to the phase I clinic at the MD Anderson Cancer Center (USA) and received immunotherapy (83). Among these, all 4 patients with ASPS showed a clinical benefit from use of ICIs; 2 patients had PR bordering CR lasting 8 and 12 months, and 2 achieved SD (83).

The efficacy of nivolumab (anti-PD-1 inhibitor) was reported by Kuo et al. and Paoluzzi et al.; one patient had PD after 3 months (84) and another had a slight PD after 9 cycles (37). However, nivolumab + ipilimumab (anti-CTLA-4 inhibitor) resulted in PR in a 29-year-old man with metastatic ASPS (85).

On December 2022, the US Food and Drug Administration approved atezolizumab (anti-PD-L1 inhibitor) for adult and paediatric patients ≥2 years old with unresectable or metastatic ASPS. This agent was reportedly effective in a 13-year-old girl with multiple brain metastases; the radiological studies suggested PD after 6 doses (15 mg/kg IV every 21 days), but pathological evaluation revealed a nonviable tumour in the resected specimens, which suggested that the Response Evaluation Criteria in Solid Tumors (RECIST) criteria may be unsuitable for evaluating ICI efficacy (86).

Recently, promising results of a phase II trial of durvalumab (anti-PD-L1 inhibitor) + tremelimumab (anti-CTLA-4 inhibitor) were reported; the overall response rates in 10 patients with ASPS by immune-related RECIST and immune-related response criteria were 40% and 50%, respectively, with 2 patients reported to have a CR (87). Somaiah et al. confirmed pseudoprogression in a few patients in this clinical trial, which was most prominent in patients with ASPS, highlighting the need for a longer duration of therapy and confirmatory scans (87).

### Combined therapy

In addition to ICI combination therapy, several clinical trials using the combined regimen of a targeted agent and an ICI have shown promising results. In a phase Ib/II trial of sunitinib (multitargeted receptor TKI) + nivolumab (anti-PD-1 inhibitor), 4 (57%) of 7 patients with ASPS had a PR (88). In a phase II trial of axitinib (anti-VEGF receptor TKI) + pembrolizumab (anti-PD-1 inhibitor) in patients with advanced sarcomas, 6 of 11 patients with ASPS achieved a PR (54.5%) and 2 of 11 (18%) achieved SD; the proportion of patients who achieved a clinical benefit was 72.7% (*n* = 8/11) (76). The median time to PR in patients was 25.1 weeks (76). Therefore, a combination of VEGF and PD-1 blockade appears feasible and promising in patients with advanced/metastatic ASPS (76).

Ongoing clinical trials are exploring the efficacy and safety of targeted agents (such as bevacizumab and selinexor, an XPO1 inhibitor) and ICIs (such as atezolizumab and nivolumab) given alone or in combinations of these agents (Table 3). These trials may reinforce prior studies of TKIs and ICIs and establish the role of these combined therapies in patients with ASPS.

**Table 3:** Ongoing (recruiting) clinical trials in alveolar soft part sarcoma (93)

Trial number	Drug	Phase	Disease	Primary endpoint	Status	Estimated enrolment	Completion date	Country
NCT03141684	Atezolizumab alone or atezolizumab + bevacizumab	II	Advanced/metastatic ASPS	ORR	Recruiting	63	31-Oct-23	USA
NCT03277924	Sunitinib and/or nivolumab + chemotherapy	I/II	Advanced bone sarcomas and STSs	PFS	Recruiting	270	30-Jun-25	Italy, Spain, UK
NCT04332874	Pembrolizumab + isolated limb infusion using melphalan and dactinomycin	II	Advanced/metastatic extremity sarcoma	PFS	Recruiting	30	1-Apr-24	USA
NCT05333458	Atezolizumab with or without selinexor	II	Unresectable/metastatic ASPS	ORR	Recruiting	77	1-May-25	USA

*Abbreviations*: ASPS, alveolar soft part sarcoma; ORR, objective response rate; STS, soft-tissue sarcoma; PFS, progression-free survival.

## Summary and future perspectives

ASPS is a unique form of an ultra-rare sarcoma that is characterized by slow progression, a high rate of distant metastasis at presentation, and resistance to conventional cytotoxic chemotherapy. A relatively higher rate of metastasis to the brain means that clinicians should include intracranial imaging in their routine imaging studies. The overall cure rate remains unsatisfactory because of the greater metastatic potential of ASPS than of other STSs. However, an overall trend towards improved survival in patients with advanced/metastatic ASPS after introducing TKIs and ICIs supports continuing efforts to develop novel therapeutic options. Given the indolent behaviour of the tumour, clinical trials of the combination of targeted therapy and immunotherapy potentially can provide evidence that these treatments can further prolong survival in patients with this type of ultra-rare sarcoma.

## Funding

This study was supported by JSPS KAKENHI Grant Numbers 21K16709 and 22H03202.

## Conflict of interest statement

None declared.
